# Bovine herpesvirus 4 glycoprotein B is indispensable for lytic replication and irreplaceable by VSVg

**DOI:** 10.1186/1746-6148-9-6

**Published:** 2013-01-09

**Authors:** Valentina Franceschi, Antonio Capocefalo, Sandro Cavirani, Gaetano Donofrio

**Affiliations:** 1 Dipartimento di Scienze Medico Veterinarie, Università di Parma, via del Taglio 10, 43126, Parma, Italy

**Keywords:** Bovine herpesvirus 4, Glycoprotein B, Homologous recombination, Gene deletion, Vesicular stomatitis virus glycoprotein

## Abstract

**Background:**

Bovine herpesvirus 4 (BoHV-4) is a gammaherpesvirus, belonging to Rhadinovirus genus, with no clear association with disease. However, there is increasing evidence of its secondary pathogenic role in cases of post-partum metritis in cattle. BoHV-4 Open Reading Frame 8 (ORF8) codifies for glycoprotein B (gB) that shows a heterodimeric structure, composed of two subunits and covalently linked by disulfide bonds and responsible for host cell adhesion through binding to heparan sulfates associated with cellular proteoglycans. Here we describe the generation of several tagged soluble forms of gB ectodomain, in order to test their ability to neutralize BoHV-4 infection.

**Results:**

The results show, however, that none of these soluble forms are able to block viral infectivity. To better understand the role of gB during BoHV-4 lytic replication, a recombinant BoHV-4 was generated by homologous recombination from a BoHV-4 cloned as a Bacterial artificial chromosome (BAC) (pBAC-BoHV-4-A), in which most of the BoHV-4 gB ORF was substituted by the insertion of a DNA stuffer selectable cassette. The resulting recombinant BoHV-4 genome (pBAC-BoHV-4-AΔgB-KanaGalK) was completely unable to reconstitute infectious replicating viral particles (Infectious Replicating Viral Particles, IRVPs) and to replicate when transfected in permissive cell lines in comparison to its revertant clone (pBAC-BoHV-4-ΔgB-Rev) or pBAC-BoHV-4-A parental clone.

**Conclusion:**

This demonstrates that the BoHV-4 replicating cycle is dependent on gB. Moreover, when gB was deleted from a recombinant BoHV-4 genome delivering an heterologous glycoprotein, Vesicular Stomatitis Virus Glycoprotein (VSVg), VSVg was unable to complement gB. This study provides direct evidence that gB is necessary for BoHV-4 lytic replication.

## Background

The Herpesvirus envelope contains a variable number of glycoproteins involved in virus attachment, penetration, budding and spreading among infected cells. Some of these proteins are extremely conserved in functions and sequences, while others are typical of a peculiar virus genus or species.

The entry model mechanism of Human Herpes Virus 1 (HHV-1) is based firstly on the interaction between glycoproteins gC and gB and the host cell heparan sulfates associated with glycosaminoglycans (GAGs) and secondly, by gD-mediated penetration that is also responsible for the fusion via gB, gH and gL [1]. Five glycoproteins are conserved in all herpesviruses, gB, gH, gL, gM and gN, and numerous studies have been carried out in order to characterize them [2].

The gB gene is one of the most conserved glycoproteins among the herpesvirus family. Homologues of gB have in fact been identified in every human and animal herpesvirus studied [3]. The hypothesis that gB function may be conserved in all herpesviruses is corroborated by its secondary structure, that shows the consistent conservation of cysteine and proline residues and the carboxy-terminal portion of the proteins [3].

Herpesvirus 4 (BoHV-4) belongs to the gammaherpesvirus, Rhadinovirus genus, and has been isolated worldwide both in healthy animals and in animals with a range of diseases varying from ocular discharge, conjunctivitis, dermatitis, respiratory diseases and abortion [4]. The pathogenic role of BoHV-4 is still unclear, even if there is increasing evidence of a secondary pathogenic role in bovine post-partum metritis [5,6].

Ten glycoproteins have been identified in BoHV-4 [7] and gB was demonstrated to be involved in cell host contact through heparan sulfate interactions [8]. It is not known if this interaction is sufficient to induce virus penetration and fusion or if a cellular receptor is specifically required, as for example occurs for Human Herpesvirus 5 (HHV-5) [9]. Structurally, gB is a heterodimeric protein, composed of two subunits linked by disulfide bonds. The protein is derived from a precursor that is first glycosylated, then trimmed and cleaved to acquire the mature gB form, thanks to a putative cleavage site which is present nearly in the middle of the sequence [3,10]. Similarly to Human Herpesvirus 4 (HHV-4), Murid Herpesvirus 4 (MuHV-4), Human Herpesvirus 8 (HHV-8) and HHV-5 [10], gB is one of the major components of the BoHV-4 virion. However the direct role of BoHV-4 gB for the progression of the virus into the lytic cycle has yet to be demonstrated. In the present study, the generation of several tagged soluble forms of BoHV-4 gB ectodomain was described, and their inability to block viral infectivity was also assessed. The cloning of different isolates of BoHV-4 genome as Bacterial artificial chromosomes (BAC) [11-13] allowed the generation of a gB-deleted mutant BoHV-4, which provided further evidence that BoHV-4 gB has an indispensable role in BoHV-4 lytic replication; moreover VSVg was unable to complement BoHV-4 gB deletion, underlying the primary role of gB during BoHV-4 life cycle.

## Methods

### DNA preparation and PCR

Virus infected cells were lysed overnight in Proteinase K Buffer containing 10 mM Tris–HCl, pH 7.5, 1 mM EDTA, 0.5% sodium dodecyl sulfate (SDS) and 100 μg of proteinase K per ml at 37°C. Nucleic acids were extracted by treatment with phenol–chloroform and precipitated with ethanol. Treatment with 100 μg/ml of RNAse A (Sigma) was performed for 1 h, after which the DNA was extracted with phenol and precipitated with ethanol again. The samples were kept at −20°C. One microgram of DNA sample was amplified over 35 cycles, each cycle consisting of denaturation at 94°C for 1 min, primer annealing at 55°C for 1 min, and chain elongation with 1 U of Pfu DNA polymerase (Fermentas) at 72°C for 2 min. PCR amplification was carried out in a final volume of 50 μl of 10 mM Tris–hydrochloride pH 8.3 containing 0.2 mM deoxynucleotide triphosphates, 3 mM MgCl_2_, 50 mM KCl, 5% DMSO and 0.25 μM of each primer. The primers used for the amplification of BoHV-4 ORF8, coding for gB protein, are SgfI-gB-sense and MluI-gB-antisense (see Table 1). The so generated 2185 bp amplicon was then checked in 1% agarose gel and visualized after ethidium bromide staining in 1× TAE buffer (40 mM Tris-acetate, 1 mM EDTA) and used to subclone gB in an expression cassette. The specificity of the PCR product was determined by sequencing.

**Table 1:** List of primers used in this work

**Primer name**	**Primer sequence 5′-3′**
SgfI-gB-sense	5^′^-CCC CCC GCG ATC GCA TGT ATT ATA AGA CTA TCT TAT TCT TCG CT-3^′^
MluI-gB-antisense	5^′^-CCC CCC ACG CGT AAG GTC TGC CAT CAT TTC AGA GAG ATC TTT-3^′^
SalI-Fc-sense	5^′^-CCC GTC GAC CGT ACG CGG CCG CTC GAG ATG CCC AGA-3^′^
BamHI-Fc-antisense	5^′^-CCC GGA TCC GCG GCC GGC CGT TTA AAC TCA TTT ACC CGG-3^′^
gB-left-sense	5^′^-CCG GAA TTC AGA ACC CAC ACT ATA GGG GAA ACG ACC TCA-3^′^
gB-left-antisense	5^′^-TCT CCA GAG GAA GAT GAT GCA CTG CAT ACT CTG-3^′^
gB-right-sense	5^′^-CCCC CTG CAG TCA CAT CCT AGA GGA ATT AAC-3^′^
gB-right-antisense	5^′^-AGA CTT GCA AGC TTC TGT GTG TAG TAA TTA-3^′^
SmaI-AseI-Kana-sense	5^′^-AAC CCC CGG GAT TAA TCC GGA ATT GCC AGC TGG GG-3^′^
SmaI-MluI-Kana anti	5^′^-CCA ACC CGG GAC GCG TGA AAT TGT AAG CGT TAA TAA T-3^′^

### Plasmids

To generate pgBFc, BoHV-4 gB ORF (from 1–2175 nt) was amplified by PCR using SgfI-gB-sense and MluI-gB-antisense primer (see Table 1), cut with SgfI/MluI and inserted in pCMV6-AC-Fc (OriGene) cut with the same enzymes. gB/Fc ORF was cut from pgB/Fc firstly with BamHI, blunt-ended, consequently cut with PmeI and then sub-cloned in pWPI (Addgene; http://www.addgene.org/12254/) cut with PmeI. Thus pWPI/gBFc was obtained.

The chimeric ORF (1510 bp) coding for gB_truncated-_gD_106_ was obtained by gene synthesis (Eurofins MWG Operon) where an NheI and a BamHI restriction sites were included to the 5^′^ and 3^′^ end respectively. This gB_truncated_gD_106_ ORF was excised from the vector (pBluescript) by NheI/BamHI digestion and inserted in pEGFP-C1 (Clontech) depleted of Green Fluorescent Protein (GFP) after cutting with the same enzymes and pCMV-gB_truncated_gD_106_ was obtained. Murine Fc was then amplified by PCR from pCMV6-AC-Fc vector with SalI-Fc-sense and BamHI-Fc-antisense primers (see Table 1) and used to substitute the gD_106_ tag in pCMV-gB_truncated_gD_106._

pCMV-gBtruncated-gD_106_ was then cut with NdeI and SalI and the gB_truncated_ was exchanged with the full length 2614 bp gB ectodomain, excised from pgB/Fc, cut with NdeI and XhoI. SalI and XhoI are compatible enzymes and the generated ends are in frame. The so generated pCMVgBgD_106_ was then cut with BamHI, blunt-ended and the fusion peptide gBgD_106_ was inserted in pWPI opened with PmeI and pWPI/gBgD_106_was generated.

pBluescript/gB_truncated_gD_106_ was cut with NheI and BamHI, the fragment was blunt-ended and inserted in PmeI cut pWPI to generate pWPI/gB_truncated_gD_106_.

pCMV-gB_truncated_gD_106_ was cut with SalI/BamHI to excise gD_106_ that was replaced with the 752bp SalI/BamHI cut murine Fc amplicon for generating pCMV-gB_truncated_Fc.

The gB_truncated_Fc was at the end excised from pCMV-gB_truncated_Fc, cut with NheI/BamHI, blunt-ended and inserted in PmeI cut pWPI to obtain pWPI/gB_truncated_Fc.

pTZ-KanaGalK, was generated by sub-cloning the 2232 bp galactokinase prokaryotic expression cassette (GalK), along with the kanamycin resistant expression cassette (Kana), into the pTZ57R shuttle vector, cut with KpnI/PstI [14]. The targeting vector, pgB-KanaGalK-gB, was generated firstly by the insertion of the 1167bp left gB homology region amplicon (gB-left-sense and antisense, see Table 1) cut with EcoRI/KpnI, in pTZ-KanaGalK, cut with the same enzymes; in this intermediate construct, cut with PstI/HindIII, was consequently subcloned the 600bp right gB homology region amplicon (gB-right-sense and antisense; see Table 1), cut with the same enzymes.

### Cells, cell culture electroporation and recombinant virus reconstitution

Bovine Embryo Kidney [(BEK) from M. Ferrari, Istituto Zooprofilattico Sperimentale, Brescia, Italy], MDBK (Madin Darby Bovine Kidney cells), BEK/cre [12], HEK (Human Embryo Kydney) and BESC (Bovine endometrial stromal cells) cell lines were cultured in Dulbecco’s modified Eagle Minimal Essential Medium (DMEM) (Lonza) containing 10% fetal bovine serum (FBS), 2 mM l-glutamine, 100 IU/ml penicillin (SIGMA) and 100 μg/ml streptomycin (SIGMA).

Cells were sub cultured to a fresh culture vessel when growth reached 70 to 90% confluence (i.e., every 3 to 5 days) and were incubated at 37°C in a humidified atmosphere of 95% air–5% CO2.

Plasmid DNAs (5 μg) in 500 μl DMEM without serum were electroporated (Equibio apparatus, 270 V, 960 μF, 4-mm gap cuvettes) into BEK or BEK/cre cells from a confluent 25-cm^2^ flask. Electroporated cells were then returned to the flask, fed the next day, and split 1:2 when they reached confluence at 2 days post electroporation. Cells were left to grow until Cytophatic Effect (CPE) appeared. Recombinant viruses were propagated by infecting confluent monolayers of MDBK cells at a multiplicity of infection (m.o.i.) of 0.5 50% tissue culture infectious doses (TCID50) per cell and maintaining them in MEM with 10% FBS for 2 h. The medium was removed and replaced by fresh MEM containing 10% FBS. When approximately 90% of the cell monolayer exhibited CPE (~72 h post infection), the virus was prepared by freezing and thawing cells three times and pelleting virions through 30% sucrose, as described previously [12]. Virus pellets were resuspended in cold MEM without FBS. TCID50 were determined on MDBK or BEK cells by limiting dilution.

### Viruses

BoHV-4-A, BoHV-4-A-ΔTK-EGFP, BAC-BoHV-4-A, BACBoHV-4-AΔL1.7VSVG, BAC-BoHV-4-AΔL1.7VSVGΔgB-KanaGalK, and BAC-BoHV-4-A-ΔgB-KanaGalK were propagated by infecting confluent monolayers of MDBK or BEK cells at a m.o.i. of 0.5 and maintained in minimal essential medium (MEM) (SIGMA) with 2% FBS for 2 h. The medium was then removed and replaced by fresh MEM containing 10% FBS. When approximately 90% of the cell monolayer exhibited CPE (approximately 72 h post infection), the virus was prepared by freezing and thawing cells three times and pelleting the virions through 30% sucrose, as previously described [12]. Virus pellets were resuspended in cold MEM without FBS. TCID50 were determined in MDBK cells by limiting dilution.

### *In vitro* neutralization assay (NTA)

HEK cells were seeded in a 175 cm^2^ flask and when reached the 85% of confluence, were transiently transfected with the plasmids carrying the gB tagged constructs.

In particular, 14 ml of DMEM without serum were incubated with 43,75 μl of LTX Lipofectamine (Invitrogen) and 17,5 μg of pWPI/gBFc, pWPI/gB_truncated_Fc, or pWPI/gB_truncated_gD_106_ at room temperature for at least 15 minutes. This transfection solution was then added carefully to the HEK cell monolayer and left to incubate 6 hours at 37°C with 5% CO_2_. The transfection solution was then removed and replaced by 21 ml of 1:1 DMEM/F12 medium without serum, after a washing, to remove any traces of the transfection solution. After 96 hours the supernatant was collected, clarified at 3000 r.p.m. for 5 minutes and stored at −20°C.

The expression of gB was assessed by Western Blotting ( the amount of gB was estimated to be between 5 and 50 μg/ml, depending on the preparation) and consequently the supernatant was used for the neutralization assay.

10^5^ HEK cells/well were seeded in a 12 multiwells plate and left to attach to the well.

After few hours the cells were pre-incubated 30 minutes at 37°C with 1ml/well of the supernatant recovered from HEK transfected with pWPI/gBFc or pWPI/gB_truncated_gD_106_ or pWPI/gB_truncated_Fc with medium without serum. The supernatant was then removed and substituted with ten-fold BoHV-4-A-ΔTK-EGFP virus dilution, starting from 1 to 10^-3^ m.o.i. The cells were then incubated overnight at 37°C in 5% humidified atmosphere.

This assay was also repeated in BESC cells, with the same protocol, but seeding 3×10^3^ cells/well in a 96 multiwell plate; the cells were then treated with the gB supernatant and infected with two-fold dilutions of the virus, starting from 10 m.o.i.

These two protocols were also tested using a contact time varying from 30 to 60 minutes and/or an incubation contact temperature of 4°C or 37°C.

The virus spreading was then monitored during the following days by fluorescence and contrast microscope analysis.

### Recombineering and selection

Recombineering was performed as previously described [15] with some modifications. Five hundred μl of a 32°C overnight culture of SW102 containing BAC-BoHV-4-A, and BAC-BoHV-4-AΔL1.7-VSVG were diluted in 25 ml Luria–Bertani (LB) medium with or without chloramphenicol (SIGMA) selection (12.5 μg/ml) in a 50 ml baffled conical flask and grown at 32°C in a shaking water bath to an OD_600_ of 0.6. Then, 12 ml were transferred to another baffled 50 ml conical flask and heat-shocked at 42°C for exactly 15 minutes in a shaking water bath. The remaining culture was left at 32°C as the un-induced control. After 15 minutes the two samples, induced and un-induced, were briefly cooled in ice/water bath slurry and then transferred to two 15 ml Falcon tubes and pelleted using 5000 r.p.m. (Eppendorf centrifuge) at 0°C for 5 min. The supernatant was poured off and the pellet was resuspended in 1 ml ice-cold ddH_2_O by gently swirling the tubes in ice/water bath slurry. Subsequently, 9 ml ice-cold ddH_2_O was added and the samples pelleted again. This step was repeated once more, the supernatant was removed and the pellet (50 μl each) was kept on ice until electroporated with gel-purified ~3.8 kb fragment (gB-KanaGalK-gB) obtained by cutting pgB-KanaGalK-gB with EcoRI/HindIII (Fermentas). An aliquot of 25 μl was used for each electroporation in a 0.1 cm cuvette at 25 μF, 2.5 kV and 201 Ω. After electroporation, the bacteria were recovered in 1 ml LB (15 ml Falcon tube) for 1 hour in a 32°C shaking water bath. For the counter selection step (see below), the bacteria were recovered in 10 ml LB in a 50 ml baffled conical flask and incubated for 4.5 hours in a 32°C shaking water bath.

After the recovery period, bacteria were washed twice in sterile 1x M9 salts (6 g/l Na_2_HPO_4_, 3 g/l KH_2_PO_4_, 1 g/l NH_4_Cl , 0.5 g/l NaCl,) (SIGMA) as follows: 1 ml culture was pelleted in an eppendorf tube at 13,200 r.p.m. for 15 seconds and the supernatant was removed with a pipette. The pellet was resuspended in 1 ml of 1× M9 salts, and pelleted again. This washing step was repeated once more. After the second wash, the supernatant was removed and the pellet was resuspended in 1 ml of 1× M9 salts before plating serial dilutions (100 μl each of 1:10, 1:100 and 1:1000 dilutions) on M63 minimal medium plates [15 g/l agar (DIFCO, BD Biosciences), 0.2% D-galactose (SIGMA), 1 mg/l D-biotin (SIGMA), 45 mg/l L-leucine (SIGMA) and 50 mg/l kanamycin (SIGMA)]. Washing in M9 salts is necessary to remove any rich media from the bacteria prior to selection on minimal medium plates. Plates were incubated 3–5 days at 32°C. Several selected colonies were picked, streaked on McConkey agar indicator plates (DIFCO, BD Biosciences) containing 50 μg/ml of kanamycin and incubated at 32°C for 3 days until red colonies appeared. Red colonies were grown overnight in 5 ml of LB containing 50 μg/ml of kanamycin and BAC-BoHV-4-AΔgB-KanaGalK were purified and analysed trough HindIII restriction enzyme digestion for gB-KanaGalK-gB fragment targeted integration into the BoHV-4-A gB locus.

SW102 bacteria containing BAC-BoHV-4-AΔgB-KanaGalK genome were also grown, heat induced as described above and electroporated with a ~2.9kb gel purified fragment (gBlocus) obtained by amplifying the gB locus with the primers gB-left-sense and gB-right-antisense. For the counter selection step, the bacteria were recovered in 10 ml LB in a 50 ml baffled conical flask and incubated for 4.5 hours in a 32°C shaking water bath. Bacterial serial dilutions were plated on M63 minimal medium plates containing 15 g/l agar, 0.2% glycerol (SIGMA), 1 mg/l D-biotin, 45 mg/l L-leucine, 0.2% 2-deoxy-galactose (DOG, SIGMA) and 12.5 μg/ml chloramphenicol. Plates were incubated for 3–5 days at 32°C. Several selected colonies were picked up, streaked on McConkey agar indicator plates (DIFCO, BD Biosciences) containing 12.5 μg/ml of chloramphenicol and incubated at 32°C for 3 days until white colonies appeared. White colonies were grown in duplicate for 5–8 hours in 1 ml of LB containing 50 μg/ml of kanamycin or LB containing 12.5 μg/ml of chloramphenicol. Only those colonies growing on chloramphenicol and not on kanamycin were kept and grown overnight in 5 ml of LB containing 12.5 μg/ml of chloramphenicol. BAC-BoHV-4-gB_revertant_ was purified and analysed through HindIII restriction enzyme digestion for gB locus fragment targeted integration. Original detailed protocols for recombineering can also be found at the recombineering website [16].

### Restriction enzyme analysis and non isotopic Southern hybridization

Fifteen μl of DNA prepared from bacteria containing pBAC-BoHV-4-A and derivatives were restriction enzyme-digested with HindIII, separated by electrophoresis overnight in a 0.8% agarose gel, stained with ethidium bromide, capillary transferred to a positively charged nylon membrane (Roche), and cross-linked by UV irradiation by standard procedures. The membrane was pre-hybridized in 50 ml of hybridization solution (7% SDS, 0.5 M phosphate, pH 7.2, 1 mM EDTA) for 2 hours at 65°C in a rotating hybridization oven (Techna instruments). Probe preparation and digoxigenin non-isotopic labelling was performed by PCR.

Southern Blotting probe was designed spanning Kana region using the primer pair SmaI-AseI-Kana sense and SmaI-MluI-Kana anti (see Table 1).

PCR amplification was carried out in a final volume of 50 μl of 10 mM Tris–HCl, pH 8.3, containing 0.2 mM deoxynucleotide triphosphates, 0.02 mM alkaline labile digoxigenin-dUTP (Roche), 3 mM MgCl_2_, 50 mM KCl, and 0.25 μM of each primer over 35 cycles, each cycle consisting of denaturation at 94°C for 1 minute, primer annealing at 55°C for 1 minute, and chain elongation with 1 U of Taq polymerase (Boehringer Diagnostics) at 72°C for 1 minute. A parallel reaction omitting digoxigenin-dUTP was performed, because digoxigenin incorporation into the amplicon can be checked through the size shift of the amplicon by gel electrophoresis. Five μl of the probe were added to 100 μl of ddH_2_O into a screw-cap tube, denatured in boiling water for 5 minutes, and cooled down on ice for another 2 minutes. Denatured probe was added to 50 ml of pre-heated 65°C hybridization solution to the pre-hybridized membrane and hybridized overnight at 65°C in a rotating hybridization oven (Techna Instruments). Following hybridization, the membrane was washed twice for 30 minutes with 100 ml of washing solution I (0.5× SSC [1× SSC is 0.15 M NaCl plus 0.015 M sodium citrate] and 0.1% SDS) and twice for 30 minutes with 100 ml of washing solution II (40 mM phosphate, pH 7.2, 0.05% SDS) at 65°C. On a freshly washed dish, the membrane was incubated for 30 min at room temperature in 25 ml of blocking solution (100 mM maleic acid, pH 7.5, 150 mM NaCl, 1% blocking reagent [Roche] or 5% skim milk). Anti-digoxigenin Fab fragment (150 U/200 μl [Roche]), diluted 1:15,000 in 25 ml of blocking solution, was applied to the membrane for 30 minutes under gentle shaking at room temperature and washed twice for 15 minutes with 100 ml of washing solution (100 mM maleic acid, pH 7.5, 150 mM NaCl, 0.3% Tween 20). Detection was performed following equilibration of the membrane in detection buffer (100 mM Tris–HCl, pH 9.5, 1 mM EDTA) for 2 minutes at room temperature. Chemiluminescent substrate (CSPD, Roche) was added by scattering the drops over the surface of the membrane after placement of the membrane between two plastic sheets, and any bubbles present under the sheet were eliminated with a damp lab tissue to create a liquid seal around the membrane. Signal detection was obtained, exposing the membrane to X-ray film. The exposure time was adjusted with the intensity of the signal.

### Western immunoblotting

Cell lysates containing 50 μg of total protein were electrophoresed through 8 to 12% SDS-polyacrylamide gels and transferred to nylon membranes by electro blotting. Membranes were incubated with monoclonal anti-BoHV-1-gD antibody (clone 1B8F11; VRMD, Inc., Pullman, WA), probed with horseradish peroxidase-labelled anti-mouse immunoglobulin G (IgG) antibody (SIGMA), and visualized by enhanced chemiluminescence (Millipore). For detecting the Fc was used an anti-mouse IgG whole molecule (A9044, Sigma, Saint Louis, Missouri).

## Results and discussion

### Soluble gB fragments did not inhibit virus attachment and replication

**Figure 1:**
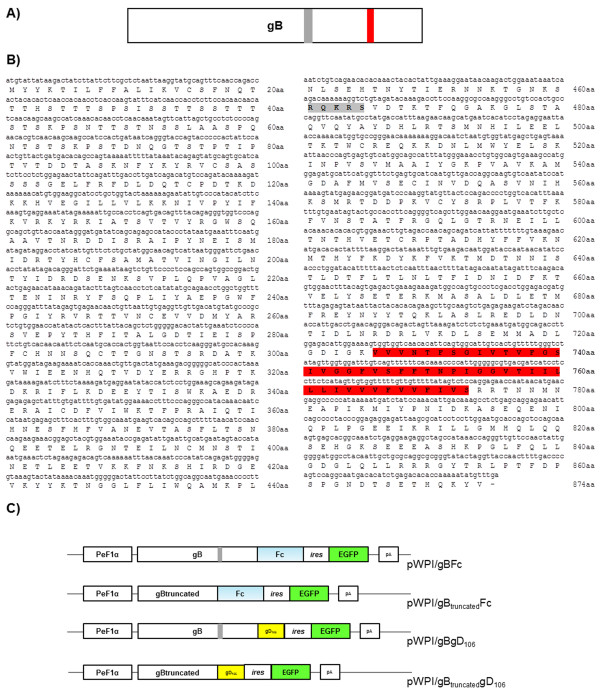
**BoHV-4 gB constructs design. A)** Schematic representation (not to scale) of BoHV-4 gB ORF; in grey is represented the putative protease site and in red the transmembrane domain. **B)** BoHV-4 gB annotated sequence with deduced amino acidic sequence, from the ATG to stop site. In grey is underlined the putative protease site (*RQKRS*) and in red the transmembrane domain. **C)** Diagram (not to scale) showing the pWPI/gBFc vector containing: the human elongation factor 1α (PeF1 α) promoter, the chimeric gB ORF ectodomain (gB) (1-720aa), the protease site is evidenced in grey, fused with the murine Fc (in sky blue), then an IRES and the EGFP ORF (in green). Diagram (not to scale) showing the pWPI/gB_truncated_Fc vector containing: the human elongation factor 1α (PeF1 α) promoter, the chimeric gB ORF ectodomain (1-420aa), fused with the murine Fc (in sky blue), then an IRES and the EGFP ORF. Diagram (not to scale) showing the pWPI/gBgD_106_ vector containing: the human elongation factor 1α (PeF1 α) promoter, the chimeric gB ORF ectodomain (1-720aa), the protease site is evidenced in grey, fused with the gD_106_ tag (in yellow), then an IRES and the EGFP ORF. Diagram (not to scale) showing the pWPI/gB_truncated_-gD_106_ vector containing: the human elongation factor 1α (PeF1 α) promoter, the chimeric gB ORF ectodomain (1-420aa), fused with the gD_106_ tag (in yellow), then an IRES and the EGFP ORF.

Recent studies have demonstrated that HHV-4 infection can be prevented by treating cells with a library of peptides homologous to the ectodomain of the HHV-4 gB. [9]. To verify if BoHV-4 gB could act as a soluble ligand for a putative BoHV-4 host cell receptor and block BoHV-4 infection, BoHV-4 gB ectodomain was sub-cloned and expressed as a secreted form into the supernatant of transiently transfected cells. BoHV-4 gB has a length of 873 aa and corresponds to an ORF of 2622 bp (Figure 1A and B). Several studies have demonstrated that the mature form of gB is heterodimeric and is generated by the presence of an internal putative proteasic site (*RQKRS*) in the second half of its aminoacidic sequence that is responsible for the cleavage of the two subunits, that are then covalently linked by disulfide bonds (Figure 1B) [3,10].

**Figure 2:**
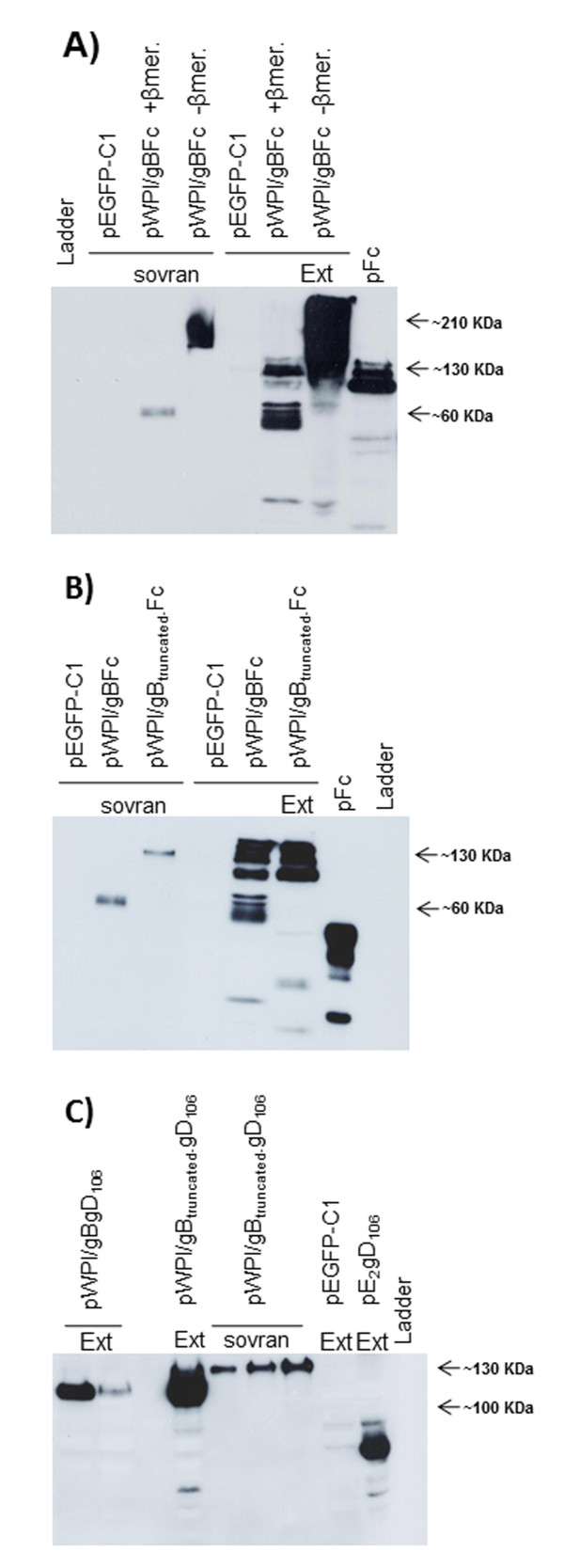
**BoHV-4 gB chimeric peptide expression. A)** Western Immunoblotting of pWPI/gBFc transfected HEK293 cell lysate or supernatant, in presence or absence of a reducing agent, β-mercaptoethanol. The cell extract of pEGFP-C1 transfected HEK 293 was used as a negative control. As a positive control, the supernatant of cells transfected with a plasmid carrying only the murine Fc was used. **B)** Western Immunoblotting of pWPI/gBFc and pWPI/gB_truncated_Fc transfected HEK293 cell lysates with the presence of β-mercaptoethanol. **C)** Western Immunoblotting of pWPI/gBgD_106_ and pWPI/gB_truncated_gD_106_ transfected HEK293 cell lysates and supernatant . The control used was the same of the Figure 2A .

The secreted forms of gB were obtained on the basis of its transmembrane domain and cytoplasmic tail exclusion and/or substitution. Firstly, the full gB ectodomain (720 aa) was fused to a murine Fc tag encoding the hinge region, the CH2 and the CH3 domains of the mouse IgG heavy chain, in order to generate gBFc (Figure 1B and Additional file 1: Figure S1A). Thus, gBFc ORF was inserted into the pWPI vector [17] and pWPI/gBFc was obtained (Figure 1B). In pWPI/gBFc, the expression of gBFc is under the control of the human elongation factor 1α promoter (EF1α). Downstream to the gBFc ORF, an internal ribosomal entry site (IRES), the GFP ORF and a Simian Virus 40 (SV40) large T antigen polyadenylation signal (pA) were also present (Figure 1B). This polycistronic construct allowed the rapid monitoring of the functionality of the construct by direct visualization of GFP expression by fluorescence microscopy (Additional file 1: Figure S1B). gBFc was correctly expressed, secreted and post-translationally modified when pWPI/gBFc was transiently transfected into HEK cells (Figure 2A and B). In fact, the variation of its size observed in western immunoblotting, was appreciable when the supernatant or protein cell extract from pWPI/gBFc transiently transfected cells were treated or untreated with reducing agents such as dithiothreitol (DTT) or β-mercaptoethanol (Figure 2A and B).

A truncated form of gB fused to Fc was also generated, gB_truncated_/Fc (Figure 1B and Additional file 2: Figure S2A), where only a part of the gB ectodomain (first 460 aa) was fused to Fc. Also in this case, gB_truncated_Fc was correctly expressed, secreted and post-translationally modified when pWPI/gB_truncated_Fc was transiently transfected into HEK cells (Figure 2B). Two other versions of gB, gBgD106 and gB_truncated_gD106 respectively, were generated replacing the Fc tag with the gD106 soluble epitope tag of BoHV-1 in order to verify the influence of the tag peptide fused with gB in terms of secretion (Figure 2B and Additional file 3: Figure S3 and Additional file 4: Figure S4).

Surprisingly, only gB_truncated_gD_106_ was efficiently secreted in transiently transfected HEK cells, as shown in Figure 2B and C. Although gBgD106 was well expressed, its secretion levels were very low. It was outside the scope of the present work to determine the reasons of the inability of gBgD_106_ to be efficiently secreted and gBgD_106_ was eliminated from the study.

**Figure 3:**
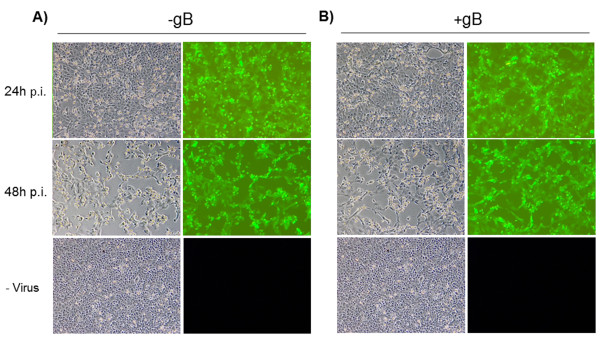
**Secreted gB neutralization assay.** Representative fluorescence and phase contrast images (10X) of MDBK cells untreated **A)** or treated **B)** for 1 hour at 37°C with BoHV-4 gB enriched supernatant and infected with one M.O.I. of BoHV-4-EGFPΔTK. Infection was monitored under a fluorescence microscopy at 24 and 48 hours after the infection. This experiment, along with others made under different condition, as indicated into the text, was repeated three times with identical results.

The supernatants containing gBFc, gB_truncated_Fc and gB_truncated_D106 were used in an *in vitro* neutralization assay (NTA) to verify if one of these secreted forms could successfully prevent BoHV-4 attachment and penetration. The NTA was performed on different cell lines (HEK, BEK, MDBK and BESC), employing a recombinant BoHV-4 expressing GFP (BoHV-4EGFPΔTK) [18], at different temperatures (37 and 4°C), time of contact (30 and 60 minutes), number of seeded cells and amount of secreted peptides. BoHV-4 attachment and penetration was monitored at different times (24 and 48 hours) by GFP expression under fluorescence microscopy. No blocking activity was achieved with any of the experimental conditions tested (Figure 3 and data not shown). The secreted gB forms tested were apparently unable to prevent BoHV-4 attachment and penetration and this was in contrast with that reported for some Herpesvirus [19], but in agreement with others [20].

The inability of gBFc, gB_truncated_Fc and gB_truncated_gD106 to prevent BoHV-4 attachment and penetration of the host cell gave rise to several hypothesis: *a)* the folding of the secreted peptide could be improper; *b)* the amount of the secreted peptide could be insufficient; *c)* viral glycoproteins different from gB could be responsible for mediating virus-cell interaction and internalization, subordinating gB to a secondary role.

### BoHV-4-AΔgB was unable to replicate in host cells

Therefore, it was of interest to investigate the above hypotheses through the disruption of the gB gene in BoHV-4 genome. The BoHV-4 gB ORF was disrupted by site specific insertional mutagenesis mediated by heat inducible homologous recombination in a strain of BoHV-4 genome cloned as a Bacterial artificial chromosome (BAC), pBAC-BoHV-4-A [12].

**Figure 4:**
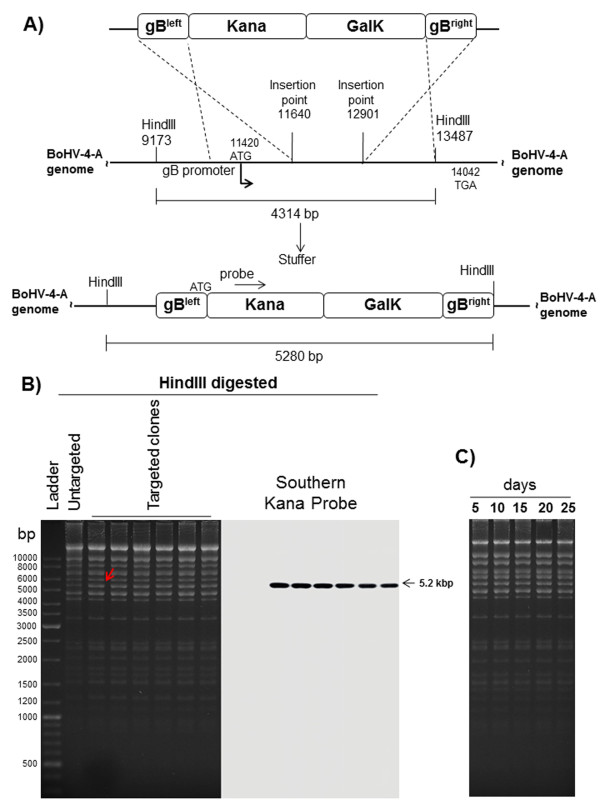
**BoHV-4 gB disruption. A)** Overall strategy to delete a 1261 bp sequence from the ORF8 coding for gB, via heat inducible homologous recombination. The 2232 bp Kana-GalK selectable DNA stuffer, flanked by ORF8 homologous regions, was introduced between positions 11640 and 12901 of the BoHV-4-A strain cloned as a BAC. The expected ORF8 locus (A, bottom) has an increased size of the HindIII fragment (5280 instead of 4314 bp), generated by HindIII restriction enzyme digestion. The diagrams here presented are not to scale. **B)** HindIII Restriction profile and corresponding Southern Blotting of six representative targeted clones, compared to the untargeted control. Southern Blotting was performed with a probe spanning Kana sequence and confirmed the above data. **C)** Clonal stability of the pBAC-BoHV-4-A-ΔgBKanaGalK in *Escherichia coli* SW102 cells, passaged for 25 consecutive days and analyzed by HindIII digestion and agarose gel electrophoresis.

**Figure 5:**
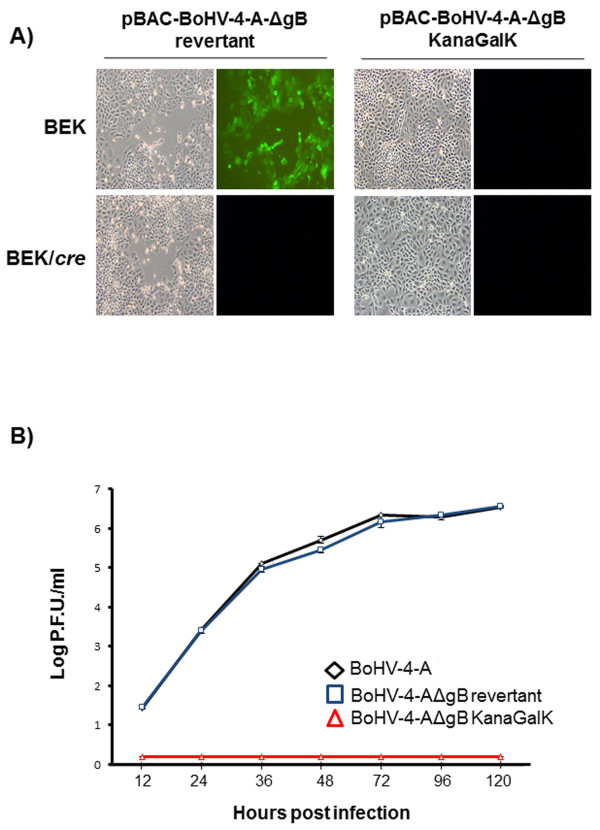
**A) Viral reconstitution.** Representative fluorescence and phase contrast images (10X) of BoHV-4-A-ΔgB_revertant_ virus reconstitution following pBAC-BoHV-4-A-ΔgB_revertant_ electroporation in BEK or BEK/cre cells. pBAC-BoHV-4-A-ΔgB_KanaGalK_ was unable to reconstitute IRVPs after electroporation in BEK or BEK/cre cells. **B)** Replication kinetics of BoHV-4-A-ΔgB_revertant_ compared with BoHV-4-A and BoHV-4-A-ΔgBKanaGalK. The data presented are the means ± standard errors of triplicate measurements.

A targeting cassette (gB-KanaGalK-gB), excised from pgB-KanaGalK-gB vector, containing the 2232 bp Kana/GalK DNA stuffer double selecting cassette [21], flanked by two BoHV-4 gB gene homology regions, was electroporated in SW102 *E.coli* containing pBAC-BoHV-4-A genome and pBAC-BoHV-4-AΔgB-KanaGalK was generated (Figure 4A). In the pBAC-BoHV-4-AΔgB-KanaGalK genome, most of the gB ORF has been replaced by Kana/GalK DNA stuffer double selection cassette (Figure 4A). All recombinant selected clones were shown to be authentic when analysed by PCR and sequencing (data not shown), HindIII restriction enzyme digestion and Southern hybridization (Figure 4B). In order to obtain the revertant genome, pBAC-BoHV-4-A-gB_revertant_, a large DNA fragment obtained by PCR from the BoHV-4-A genome, was electroporated in SW102 *E.coli* containing the pBAC-BoHV-4-AΔgB-KanaGalK genome. Following a double negative selection [12], several revertant clones were obtained as showed by PCR, sequencing (data not shown), HindIII restriction enzyme digestion and Southern hybridization (Additional file 5: Figure S5A and B). The selected clones’ stability was assessed by serially passaging over 25 days and analysis by HindIII restriction enzyme digestion (Figure 4C and Additional file 5: Figure S5C). To reconstitute infectious viruses, pBAC-BoHV-4-AΔgB-KanaGalK and pBAC-BoHV-4-A-gB_revertant_ were electroporated into BEK or BEK/cre cells to excise the BAC cassette. Surprisingly, the viable virus was obtained only from pBAC-BoHV-4-A-gB_revertant_ but not from pBAC-BoHV-4-AΔgB-KanaGalK, in both BEK and BEK/cre cells (Figure 5A). The deletion of gB rendered BoHV-4 unable to be reconstituted and productively replicated. BoHV-4-A-gB_revertant_ growth characteristics were identical to the parental BoHV-4-A (Figure 5B).

### VSVg could not complement BoHV-4 gB

Various attempts have been reported regarding gB complementation in several gammaherpesviruses using heterologous gB [20,22]. It was thus decided here to try to complement gB with VSVg, which has been previously shown to complement HSV-1 gB [20] and which is also widely used to pseudo-type lentiviral vectors [23].

BoHV-4 gB ORF was deleted as was done for pBAC-BoHV-4-AΔgB-KanaGalK, in BoHV-4-AΔL1.7-VSVG (Additional file 6: Figure S6A, B and C). BoHV-4-AΔL1.7-VSVG is a replicating competent attenuated BoHV-4 expressing VSVg, where the VSVg expression cassette is inserted into BoHV-4 L1.7 gene locus (Capocefalo et al.; *submitted paper*). When pBAC-BoHV-4-AΔL1.7-VSVGΔgB-KanaGalK was electroporated into BEK or BEK/cre it was unable to reconstitute replicating viral particles (data not shown), as was observed for the deletion of gB from BoHV-4-A. This is a further demonstration that BoHV-4 gB is essential and cannot be complemented by a heterologous protein such as VSVg.

Virus entry into host cells normally requires one glycoprotein involved in cell binding and membrane fusion, whereas herpesviruses use several viral glycoproteins to enter cells. Herpesvirus entry relies on a complex multiprotein mechanism that is not yet well understood. Even if the role of each single protein potentially involved is widely studied, their action as a complex, determining virus adhesion and cell entry has not been fully clarified.

Gammaherpesviruses can infect different cell types in a cell-dependent manner, as is the case for HHV-4, which is able to infect B cells through the action of the gp350, gp42, gB, gH and gL proteins, whereas it can infect epithelial cells only with gB, gH and gL [24]. In this complicated panorama, the role of gB is usually conserved with respect to virus adhesion and fusion to the host cells. Indeed, a gB homologue has been identified in all Gammaherpesviruses [25].

In MuHV-4, gB and gH are required for cell fusion and the N-terminal half of gB is thought to be responsible for the fusion, containing a putative fusion loop. A change in pH drives the fusion and gB changes its state from a pre-fusion state to a post-fusion one. Specific mAbs directed against gB block membrane fusion, maintain gB in its post-fusion form, block the release of virions from late endosomes [26], thus neutralizing infection. Of the ten glycoproteins that have been described in BoHV-4 [7], gB is the most abundant and one of the major components of the virion. Similarly to the majority of other gammaherpesviruses, like Equine Herpesvirus 2 (EHV-2) and 5 (EHV-5) and MuHV-4, BoHV-4 gB is present in the virion in its cleaved form [10,27]. The BoHV-4 ORF8 coding for gB is 2622 bp in length and has a strict homology with HHV-4 gB, indicating that these sequence similarities could correspond to similar secondary and tertiary structures [3]. BoHV-4 gB transcript is unspliced and shows a typical TATA box sequence positioned 80 nucleotides upstream of the first ATG and a putative polyadenylation signal can be identified in 3^′^ respect to the stop codon. BoHV-4 gB is an heterodimeric glycoprotein, called gp6-gp10-gp17, with an apparent molecular size of 150 kDa-120 kDa-51 kDa respectively. gp10-gp17 are linked by disulfide bonds and gp6 by non-covalent bonds and all these subunits derive from the proteolitical cleavage of the gB gene product [7]. BoHV-4 gB interaction with heparan-like molecules present on host cell GAGs was previously shown [8], demonstrating BoHV-4 gB contribution to virus entry and fusion, but its direct necessity has not yet been demonstrated.

## Conclusion

The present study may be considered the direct demonstration that gB is essential for BoHV-4 replication and its deletion is not compatible with virus survival. Moreover, despite the structural similarity between BoHV-4 gB and VSVg proteins, gB substitution with VSVg is not effective, emphasising the unique and specific role of gB in BoHV-4 life cycle.

## Competing interests

The authors declare that they have no competing interests.

## Authors’ contribution

VF performed the experiments and contributed to write the paper; AC contributed to perform the experiments; SC intellectually contributed; GD designed the study, interpret the data, performed the experiments and wrote the paper. All authors read and approved the final manuscript.

## Supplementary Material

Additional file 1 **Figure S1. A)** Chimeric peptide gB/Fc sequence and predicted amino-acid product. In black is highlighted the gB sequence, in grey the putative protease site, *RQKRS,* and in sky blue the Fc sequence. **B)** Representative contrast phase and fluorescence images of pWPI/gBFc transfected HEK cells at 24 hours after transfection. Click here for file

Additional file 2 **Figure S2. A)** Chimeric peptide gB_truncated_Fc sequence and predicted amino-acid product. In black is highlighted the gB truncated sequence and in sky blue the Fc sequence. **B)** Representative contrast phase and fluorescence images of pWPI/gB_truncated_Fc transfected HEK cells at 24 hours after transfection. Click here for file

Additional file 3 **Figure S3. A)** Chimeric peptide gB/gD_106_ sequence and predicted amino-acid product. In black is highlighted the gB sequence, in grey the putative protease site, *RQKRS,* in yellow the gD_106_ sequence. **B)** Representative contrast phase and fluorescence images of pWPI/gBgD_106_ transfected HEK cells at 24 hours after transfection. Click here for file

Additional file 4 **Figure S4. A)** Chimeric peptide gB_truncated_-gD_106_ sequence and predicted amino-acid product. In black is highlighted the gB truncated sequence and in yellow the gD_106_ tag sequence. **B)** Representative contrast phase and fluorescence images of pWPI/gB_truncated_-gD_106_ transfected HEK cells at 24 hours after transfection. Click here for file

Additional file 5 **Figure S5. A)** Overall strategy to reconstitute the ORF8 complete gB locus, via heat inducible homologous recombination. The 2911 bp gB locus, amplified by PCR (between positions 10576 and 13487 of BoHV-4-A genome), was introduced to replace the 2232 bp Kana-GalK selectable DNA stuffer, flanked by ORF8 homologous regions in the BoHV-4-A ΔgBKanaGalK strain cloned as a BAC. The expected ORF8 locus (A, bottom) has decreased size of the HindIII fragment (4314 instead of 5280 bp), generated by HindIII restriction enzyme digestion (diagram not on scale). **B)** HindIII restriction profile and corresponding Southern Blotting of five representative targeted clones, compared to the untargeted control. Southern Blotting was performed with a probe spanning Kana sequence and confirmed the above data. **C)** Clonal stability of the pBAC-BoHV-4-A-ΔgB_revertant_ in *Escherichia coli* SW102 cells, passaged for 25 consecutive days and analyzed by HindIII digestion and agarose gel electrophoresis. Click here for file

Additional file 6 **Figure S6.** Overall strategy to delete a 1261 bp sequence from the ORF8 codifying for gB, via heat inducible homologous recombination. The 2232 bp Kana-GalK selectable DNA stuffer, flanked by ORF8 homologous regions, was introduced between positions 11640 and 12901 of the BoHV-4-AΔL1.7-eF-VSVG strain cloned as a BAC. The expected ORF8 locus (A, bottom) has an increased size of the HindIII fragment (5280 instead of 4314 bp), generated by HindIII restriction enzyme digestion (diagram not on scale). **B)** HindIII Restriction profile and corresponding Southern Blotting of six representative targeted clones, compared to the untargeted control. Southern Blotting was performed with a probe spanning Kana sequence and confirmed the above data. **C)** Clonal stability of the pBAC-BoHV-4-A-ΔgBKanaGalK in *Escherichia coli* SW102 cells, passaged for 30 consecutive days and analyzed by HindIII digestion and agarose gel electrophoresis. Click here for file
