# Supplementary figures and images for: Bovine herpesvirus 4 glycoprotein B is indispensable for lytic replication and irreplaceable by VSVg
Source: BMC Vet Res. 2013 Jan 9;9:6. doi: 10.1186/1746-6148-9-6 (PMC3549454; doi:10.1186/1746-6148-9-6)

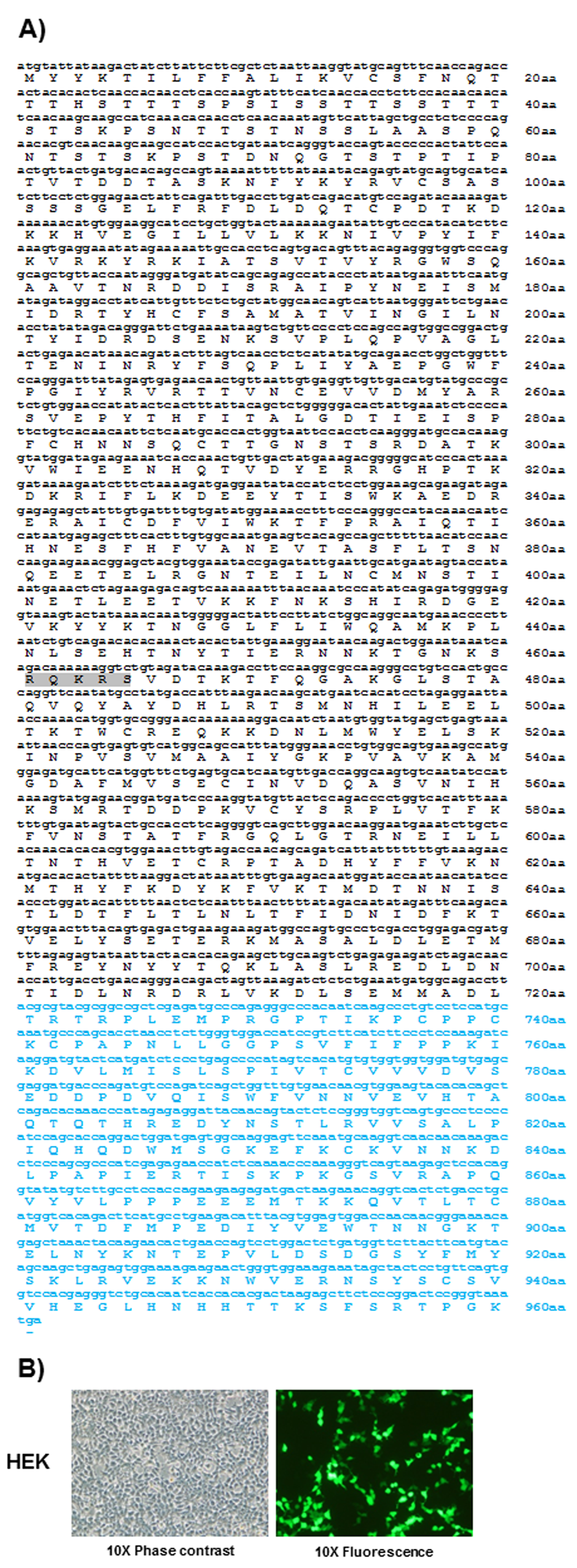

Supplement: Additional file 1 — Figure S1. A) Chimeric peptide gB/Fc sequence and predicted amino-acid product. In black is highlighted the gB sequence, in grey the putative protease site, RQKRS, and in sky blue the Fc sequence. B) Representative contrast phase and fluorescence images of pWPI/gBFc transfected HEK cells at 24 hours after transfection. [file 1746-6148-9-6-S1.tiff]

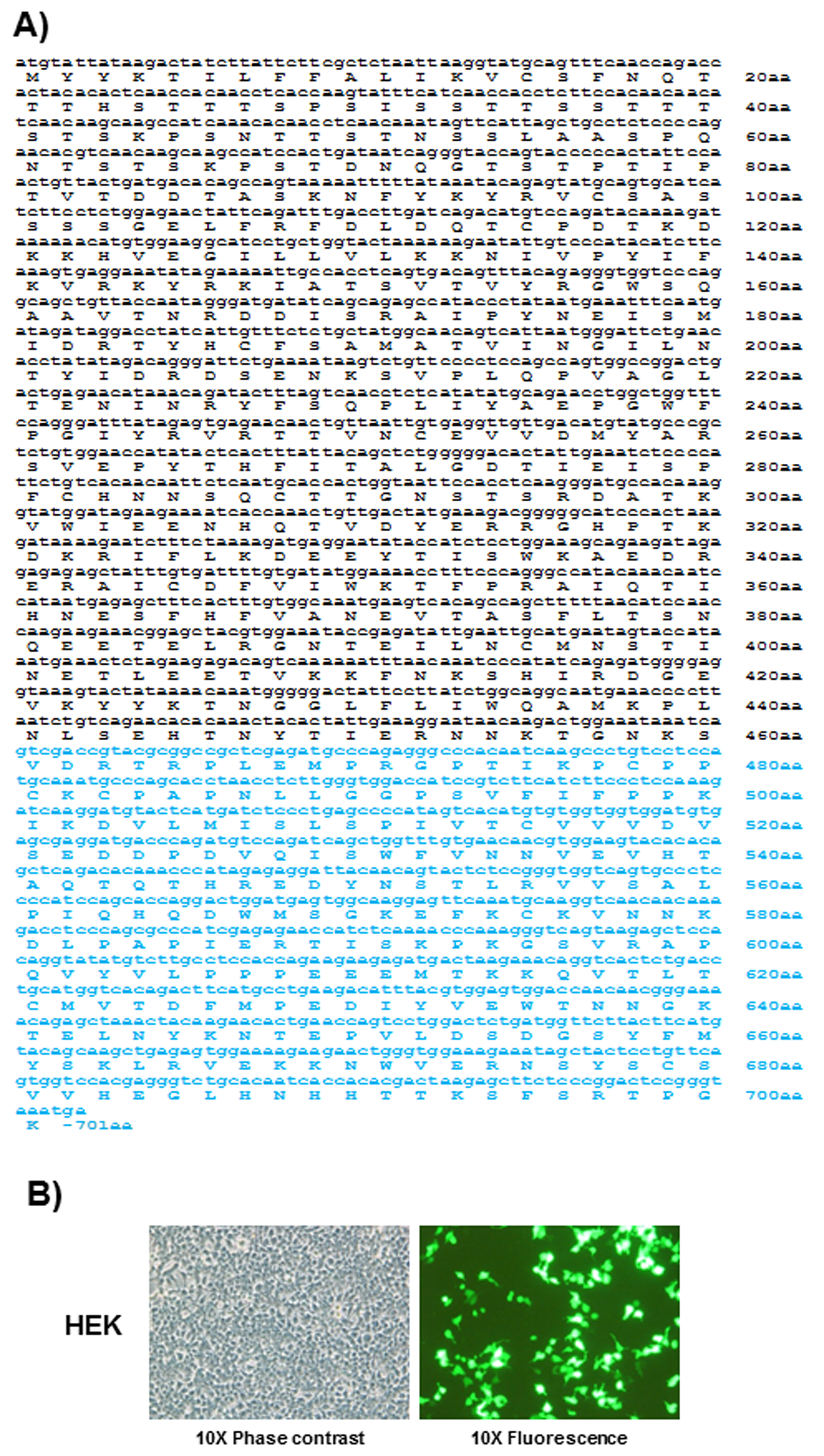

Supplement: Additional file 2 — Figure S2. A) Chimeric peptide gBtruncatedFc sequence and predicted amino-acid product. In black is highlighted the gB truncated sequence and in sky blue the Fc sequence. B) Representative contrast phase and fluorescence images of pWPI/gBtruncatedFc transfected HEK cells at 24 hours after transfection. [file 1746-6148-9-6-S2.tiff]

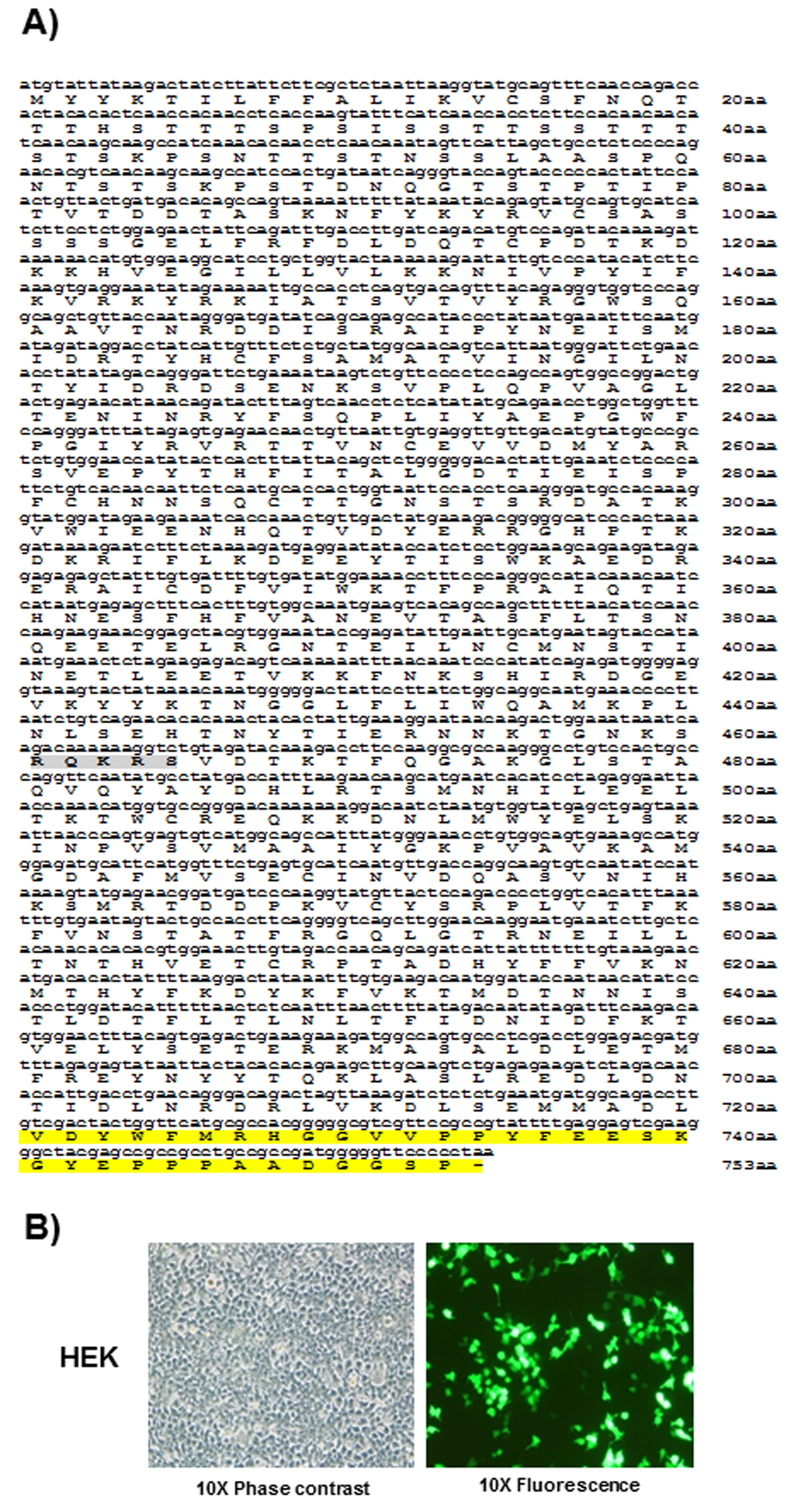

Supplement: Additional file 3 — Figure S3. A) Chimeric peptide gB/gD106 sequence and predicted amino-acid product. In black is highlighted the gB sequence, in grey the putative protease site, RQKRS, in yellow the gD106 sequence. B) Representative contrast phase and fluorescence images of pWPI/gBgD106 transfected HEK cells at 24 hours after transfection. [file 1746-6148-9-6-S3.tiff]

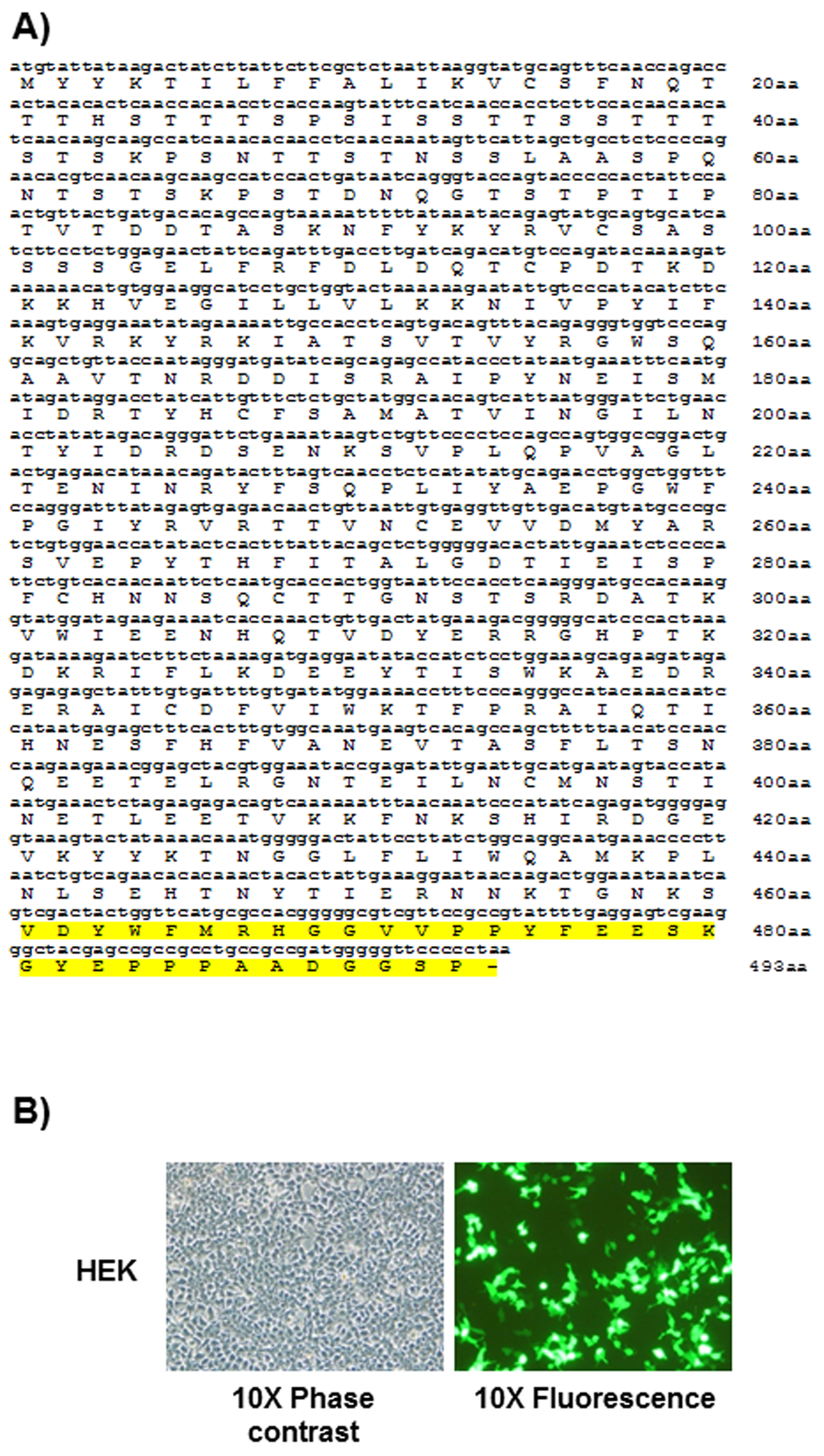

Supplement: Additional file 4 — Figure S4. A) Chimeric peptide gBtruncated-gD106 sequence and predicted amino-acid product. In black is highlighted the gB truncated sequence and in yellow the gD106 tag sequence. B) Representative contrast phase and fluorescence images of pWPI/gBtruncated-gD106 transfected HEK cells at 24 hours after transfection. [file 1746-6148-9-6-S4.tiff]

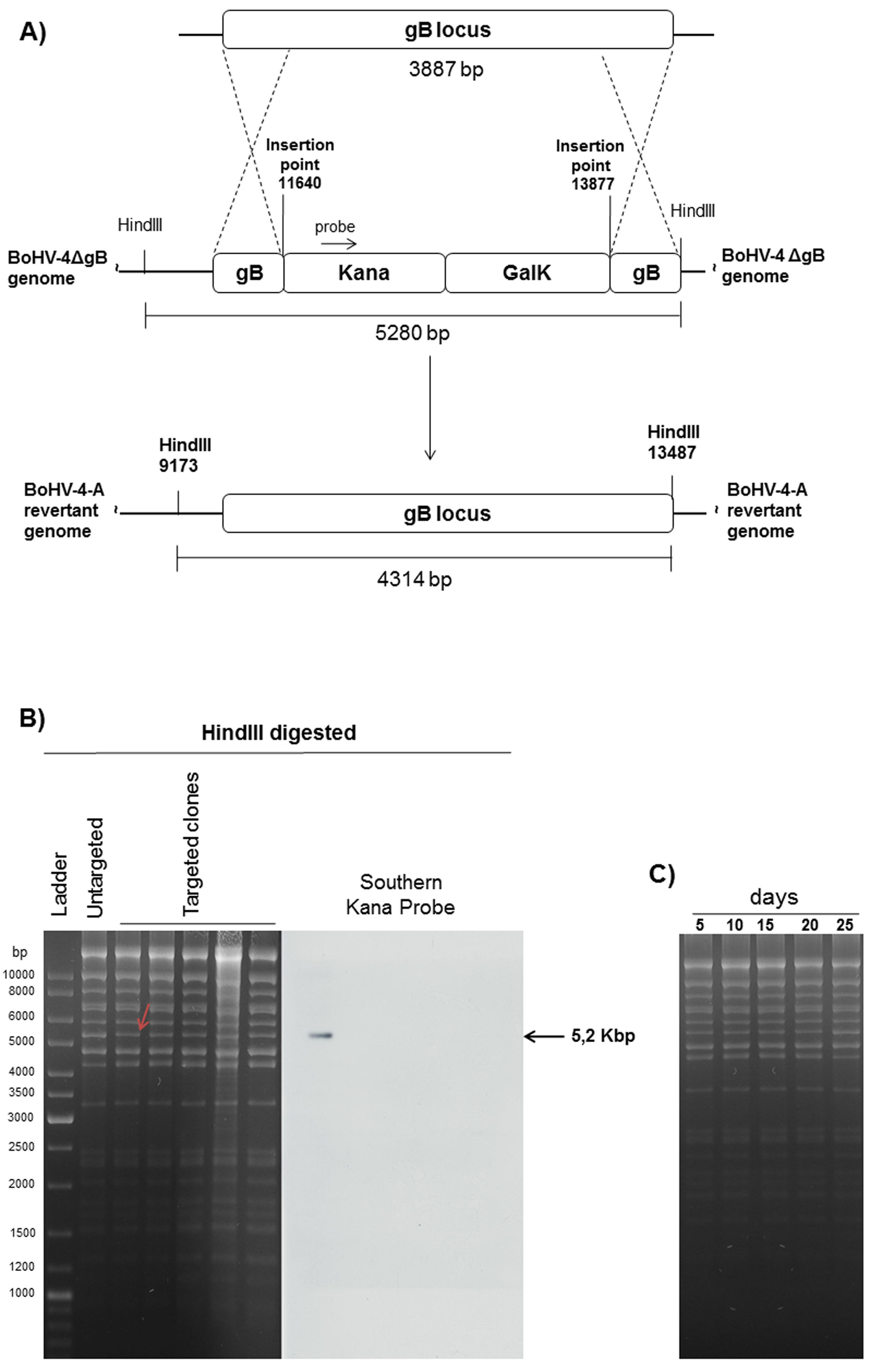

Supplement: Additional file 5 — Figure S5. A) Overall strategy to reconstitute the ORF8 complete gB locus, via heat inducible homologous recombination. The 2911 bp gB locus, amplified by PCR (between positions 10576 and 13487 of BoHV-4-A genome), was introduced to replace the 2232 bp Kana-GalK selectable DNA stuffer, flanked by ORF8 homologous regions in the BoHV-4-A ΔgBKanaGalK strain cloned as a BAC. The expected ORF8 locus (A, bottom) has decreased size of the HindIII fragment (4314 instead of 5280 bp), generated by HindIII restriction enzyme digestion (diagram not on scale). B) HindIII restriction profile and corresponding Southern Blotting of five representative targeted clones, compared to the untargeted control. Southern Blotting was performed with a probe spanning Kana sequence and confirmed the above data. C) Clonal stability of the pBAC-BoHV-4-A-ΔgBrevertant in Escherichia coli SW102 cells, passaged for 25 consecutive days and analyzed by HindIII digestion and agarose gel electrophoresis. [file 1746-6148-9-6-S5.tiff]

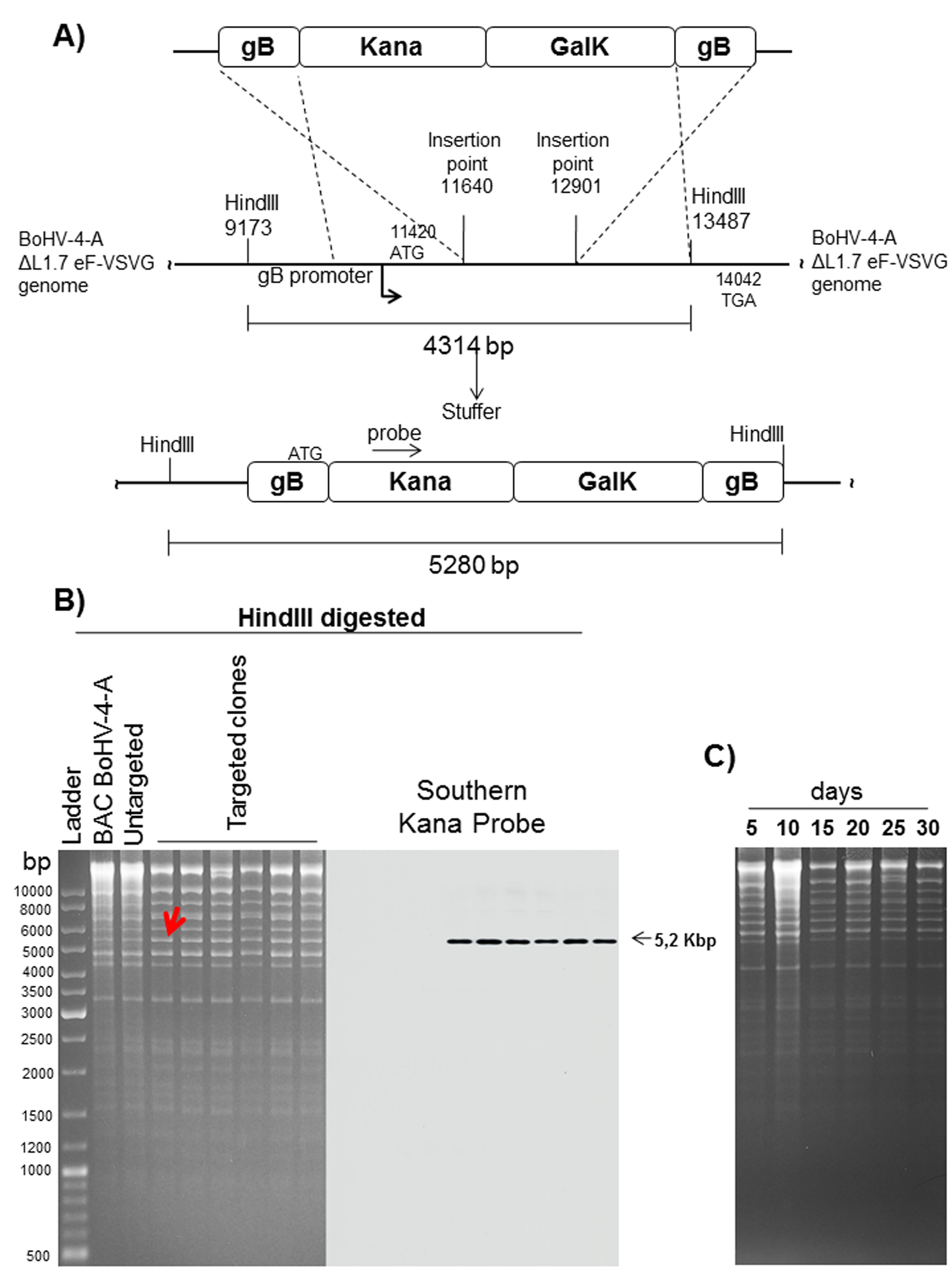

Supplement: Additional file 6 — Figure S6. Overall strategy to delete a 1261 bp sequence from the ORF8 codifying for gB, via heat inducible homologous recombination. The 2232 bp Kana-GalK selectable DNA stuffer, flanked by ORF8 homologous regions, was introduced between positions 11640 and 12901 of the BoHV-4-AΔL1.7-eF-VSVG strain cloned as a BAC. The expected ORF8 locus (A, bottom) has an increased size of the HindIII fragment (5280 instead of 4314 bp), generated by HindIII restriction enzyme digestion (diagram not on scale). B) HindIII Restriction profile and corresponding Southern Blotting of six representative targeted clones, compared to the untargeted control. Southern Blotting was performed with a probe spanning Kana sequence and confirmed the above data. C) Clonal stability of the pBAC-BoHV-4-A-ΔgBKanaGalK in Escherichia coli SW102 cells, passaged for 30 consecutive days and analyzed by HindIII digestion and agarose gel electrophoresis. [file 1746-6148-9-6-S6.tiff]
